# Reversible Dissolution of Microdomains in Detergent-Resistant Membranes at Physiological Temperature

**DOI:** 10.1371/journal.pone.0132696

**Published:** 2015-07-06

**Authors:** Andrea Cremona, Francesco Orsini, Paola A. Corsetto, Bart W. Hoogenboom, Angela M. Rizzo

**Affiliations:** 1 Dipartimento di Scienze Farmacologiche e Biomolecolari, Università degli Studi di Milano, Milano, Italy; 2 Dipartimento di Fisica, Università degli Studi di Milano, Milano, Italy; 3 London Centre for Nanotechnology, University College London, London, United Kingdom; 4 Department of Physics and Astronomy, University College London, London, United Kingdom; Institut Curie, FRANCE

## Abstract

The formation of lipid microdomains (“rafts”) is presumed to play an important role in various cellular functions, but their nature remains controversial. Here we report on microdomain formation in isolated, detergent-resistant membranes from MDA-MB-231 human breast cancer cells, studied by atomic force microscopy (AFM). Whereas microdomains were readily observed at room temperature, they shrunk in size and mostly disappeared at higher temperatures. This shrinking in microdomain size was accompanied by a gradual reduction of the height difference between the microdomains and the surrounding membrane, consistent with the behaviour expected for lipids that are laterally segregated in liquid ordered and liquid disordered domains. Immunolabeling experiments demonstrated that the microdomains contained flotillin-1, a protein associated with lipid rafts. The microdomains reversibly dissolved and reappeared, respectively, on heating to and cooling below temperatures around 37°C, which is indicative of radical changes in local membrane order close to physiological temperature.

## Introduction

A typical cell membrane is overwhelmingly complex, as it contains two asymmetric leaflets, proteins, and hundreds of lipid species, such as glycerophospholipids, sphingolipids and cholesterol, that greatly differ in their physico-chemical properties [1]. It is becoming increasingly apparent that such a complex assortment of lipids is necessary to accomplish the manifold functions that lipids perform. As originally proposed in Singer and Nicolson’s "fluid mosaic" model [2], cellular membranes appear as two-dimensional solutions of integral membrane proteins in a lipid bilayer solvent [3], allowing the lateral movement of membrane components. This fluidity does not preclude the formation of membrane domains that differ in lipid and/or protein composition from that of the surrounding lipid matrix [4].

Such domains, also called “lipid rafts”, have been postulated as dynamic, small and ordered domains enriched in cholesterol, sphingomyelin and specific proteins, which may fuse into larger structures due to lipid–lipid, lipid–protein, and protein–protein interactions [5]. This organization of membranes into dynamic platforms serves to bring about a functional compartmentalization so that multimolecular interactions, such as membrane trafficking, signal transduction, and regulation of the activity of membrane proteins, can be executed and regulated in space and time [6].

The formation of these domains may appear a natural consequence of the heterogeneous character of the cell membrane: At sufficiently low temperatures, the entropic gain of mixing different liquid membrane components can be outweighed by the enthalpic gain of demixing them into liquid ordered (Lo) and liquid disordered (Ld) domains, before–on further cooling–the lipids transit into the gel phase [7]. Using a variety of techniques, such as Fluorescence Resonance Energy Transfer, deuterium-based Nuclear Magnetic Resonance and Atomic Force Microcopy (AFM), it has been shown that lipid model membranes can indeed laterally segregate into differently sized domains [8]. As demonstrated by AFM experiments on supported lipid bilayers consisting of 1,2-Dioleoylphosphatidylcholinel (DOPC), sphingomyelin and cholesterol, phase separation occurs below a critical temperature between 30–40°C for compositions similar to those in cell membranes [9]. AFM experiments on supported membranes have the advantages of high spatial resolution and that they are label-free, provide a direct measure of the order parameter via the difference in membrane thickness of the Lo and Ld phases, and allow for accurate quantification of the different phases as a function of temperature.

Although such experiments have greatly improved our understanding of the structure and function of cell membranes, the model membranes do not reflect the full complexity of the lipid environment or the interactions between lipids and proteins in a cell membrane. Giant plasma membrane vesicles, microscopic spheres of plasma membranes harvested from live cells following chemical treatment [10] are probably the closest models of cell plasma membranes in terms of chemical composition, as in addition to lipids they contain also membrane proteins. Fluid phase separation has been reported in giant plasma membrane vesicles obtained from mast cells and fibroblasts [11,12], from A431 [13] and RBL-2H3 [14] cells. Bernardino de la Serna and coworkers [15], using fluorescence microscopy and AFM under near-physiological conditions, observed the coexistence of two distinct micrometer-sized fluid phases in giant plasma membrane vesicles obtained from native pulmonary surfactant membranes. Moreover, their results showed that the phase separation was dramatically affected by the extraction of cholesterol.

In intact cell membranes, lipid domains have been substantially harder to characterize, though in some cases ordered domains have been observed [16–20]. Alternatively, isolated, detergent-resistant membranes have traditionally been used for the study of lipid rafts. They typically have a composition that in complexity is similar to that of the plasma membranes of living cells, including lipid heterogeneity and protein contents, though enriched in cholesterol and sphingomyelin, and relatively depleted of glycerophospholipids [21, 22]. They have been isolated from a wide variety of cultured cells, including almost all the mammalian cell types [23–25], yeast [26], and plant cells [27].

Detergent-resistant membranes have traditionally been related to insoluble lipid-protein domains and associated proteins in native membranes, since detergent-lipid insolubility was found to correlate with the presence of pre-existing ordered lipid domains in native membranes [28]. However, without a clear measure of domain stability under the isolation conditions, the amount of insoluble lipid recovered after detergent treatment is unlikely to always be an accurate measure of the amount in ordered domains prior to detergent addition. In addition, detergent-resistant membranes are typically isolated at 4°C, as the low temperature allows ordered domains stabilizes their lipid-lipid and lipid-protein interactions and thereby enhances their insolubility in detergent. E.g., it has been shown that sphingomyelin-ceramide mixtures were resistant to detergent solubilization at 4°C, but became fully solubilized at 50°C [29]. This raises questions about the ordered nature of thus isolated detergent-resistant membranes when the temperature is raised to more physiological values.

Following previous studies of microdomains in isolated membranes [30–32], we here use AFM for an accurate characterization of domain size and of structural differences between different lipid phases in detergent-resistant membranes, purified from MDA-MB-231 human breast cancer cells. In addition, we have characterized their temperature dependence, to demonstrate the reversible dissolution of microdomains at physiological temperature.

## Materials and Methods

### Cell Lines and Culture Conditions

The human breast cancer cell line MDA-MB-231, (ER-negative and over-expressing EGFR), was purchased from the IST (Italian National Cancer Research Institute, Genoa, Italy, Laboratory of Molecular Mutagenesis and DNA repair). The cells were cultured in DMEM medium (Gibco-BRL, Life Technologies Italia srl, Italy), supplemented with 10% fetal bovine serum (FBS), penicillin (100U/mL), streptomycin (100mg/mL) and glutamine (2mM), and maintained at 37°C in a 5% CO_2_ atmosphere with 98% relative humidity.

### Isolation of Detergent-Resistant Membranes

Cells were harvested by scrapering in PBS containing 0.4mM Na_3_VO_4_. Next, they were centrifuged and lysed in 1.4mL ice-cold lysis buffer (1% Triton X-100, 10mM Tris buffer, pH 7.5, 150mM NaCl, 5mM EDTA, 1mM Na_3_VO_4_, 1mM phenylmethylsulfonyl fluoride, 75milliunits/ml aprotinin), in the presence of a non-ionic detergent (Triton X-100) to solubilize all membranes, and of protease inhibitors (phenylmethylsulfonyl fluoride and aprotinin) to prevent protein degradation. After 20min, the cell lysate was thoroughly homogenized by a tight-fitting dounce homogenizer, and then centrifuged at 1300g for 5min at 4°C. The resulting post-nuclear supernatant was transferred to an Eppendorf tube. Detergent-resistant membrane fractions were isolated from total cell lysate using 5–30% discontinuous sucrose gradients. Briefly, 1mL of the post-nuclear supernatant was mixed in an ultracentrifuge tube (Beckman Coulter) with 1mL of ice-cold 85% (w/v) sucrose in TNEV (10mM Tris buffer, pH 7.5, 150mM NaCl, 5mM EDTA, and 1mM Na_3_VO_4_) and then overlaid with 30% and 5% (w/v) sucrose solutions respectively. The gradient was centrifuged at 4°C for 17h at 200,000g (Beckman Coulter Optima LE-80K Ultracentrifuge, Palo Alto, CA). Eleven 1mL fractions were collected by pipette in a top down fashion, and detergent-resistant membranes were collected in low-density fractions (fractions 5 and 6) corresponding to the 5% and 30% sucrose interface. All measurements presented here were carried out on membranes from fraction 5. With this preparation, detergent is likely to still be present in the isolated membranes.

### AFM Imaging

Purified membrane samples obtained by the ultracentrifugation process were diluted 1:30 in adsorption buffer (150mM KCl, 25mM MgCl_2_, 10mM Tris/HCl pH 7.5). 50μL of the suspension was floated on a freshly cleaved mica specimen disk for 10min, at room temperature. Next, the sample was gently rinsed with a recording buffer (150mM KCl, 10mM Tris/HCl pH 7.5) to remove membranes that had not been strongly adsorbed to the mica substrate. The sample was scanned in recording buffer using MSNL-A cantilevers with nominal spring constant of 0.07N/m (Bruker, Santa Barbara, CA). All topography AFM images were collected in tapping mode using a Multimode AFM with a Nanoscope IIIa controller (Bruker), extended by separate electronics (Nanoscan, Duebendorf, Switzerland) to detect the amplitude and phase of the oscillating cantilever. The tapping frequency was about 30kHz, and the images of 512×512 pixels were recorded with a line rate of 1-2Hz. Once adsorbed to the mica substrate, membrane samples were visualized at various temperatures in the range 23–45°C, controlled with the integrated heater in the Multimode JVH scanner; using a small thermistor, it had been verified that the corresponding High Temperature Heater Controller (Bruker) indicated the actual temperature on the sample to ±1°C accuracy. Control measurements on a calibration plate confirmed the position calibration of the scanner to within 3% accuracy over the temperature range that was used in this work. AFM images were recorded at a temperature that was constant within ±0.1°C.

### AFM Data Analysis

All AFM images were line-by-line background-subtracted (flattened) and analyzed using the NanoScope analysis software (Bruker). Surface areas were determined with the same software using its bearing analysis function, which provides a method of plotting and analyzing the distribution of surface height over a sample. In particular, threshold heights of 6 nm, 5 nm and 3.5 nm were used to calculate the surface area of the bright spots visualized on the membrane surface, microdomains and membrane patches, respectively. To verify the robustness of the method, its results were compared to the areas determined by tracing the edges of microdomains and patches, and subsequently estimating the number of pixels contained within the traces. The results of the two methods were consistent to within 5%.

### AFM-Immunolabeling

Purified membrane samples were incubated with anti flotillin-1 antibody (Santa Cruz Biotechnology, USA). The antibody was diluted 1:4 in recording buffer (to 50 μg/ml), and the sample was imaged after 60min of the antibody incubation. The difference of the surface area of the membrane samples visualized in AFM images, before and after the antibody incubation, was determined following the procedures reported elsewhere [31].

## Results

**Fig 1:**
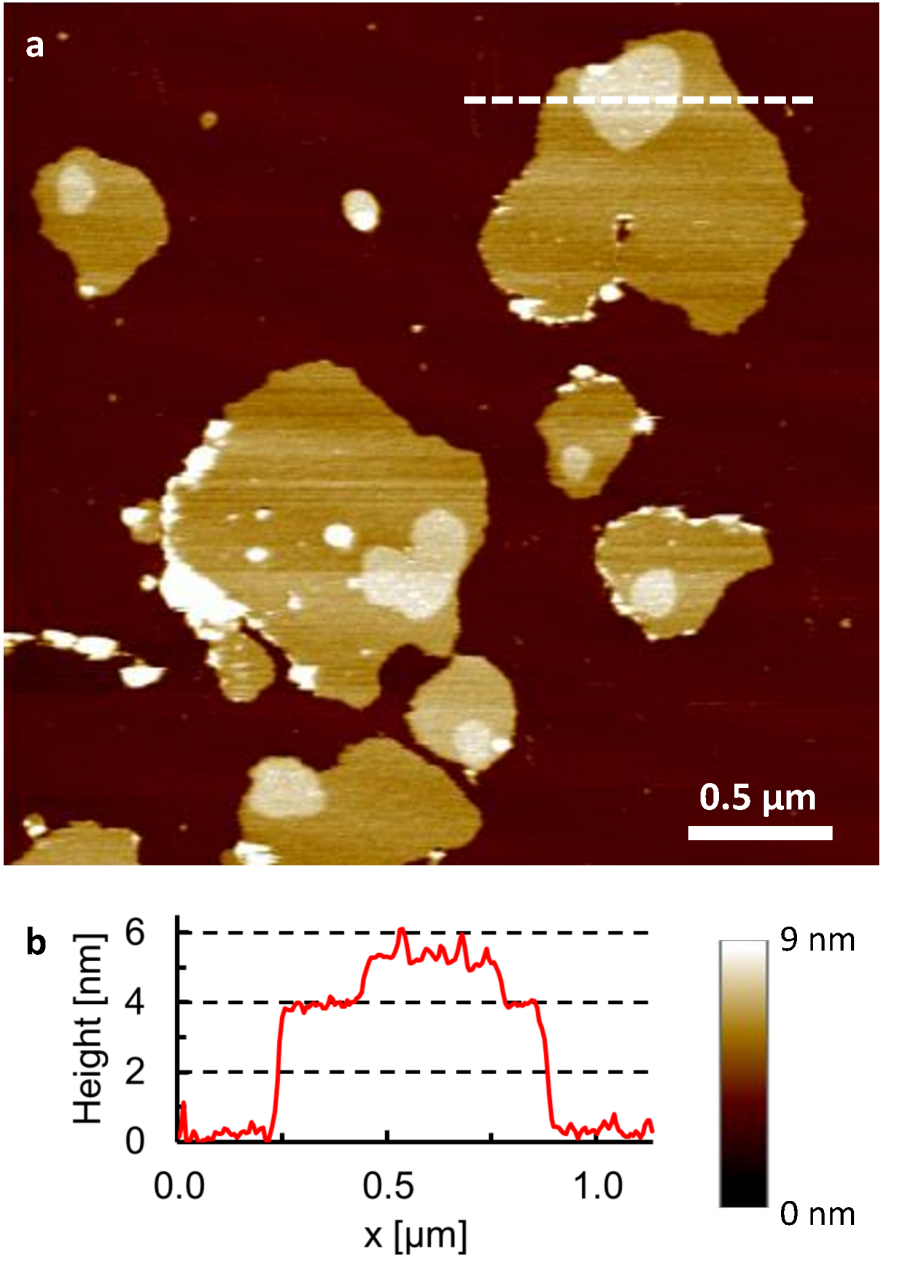
Microdomains in detergent-resistant membranes. **a**, AFM topography of detergent-resistant membranes (isolated on sucrose gradient, fraction 5) adsorbed on a mica substrate (dark brown), recorded in buffer solution at 25°C. Microdomains appear as elevated (brighter) plateaus with lateral dimensions of 100–300 nm on top of the membrane patches. **b**, Height profile corresponding to the white line drawn in (a).

Detergent-resistant membranes were purified from MDA-MB-231 human breast cancer cells by ultracentrifugation on a discontinuous sucrose gradient. For low-density fractions (fractions 5 and 6) at the 5% and 30% sucrose interface, the isolated membranes were enriched in cholesterol and sphingomyelin, as demonstrated by High-Performance Thin Layer Chromatography, and Western Blotting of these membranes (S1 Fig) demonstrated the presence of flotillin-1, a protein associated with lipid rafts [31]. The results presented here all refer to membranes from fraction 5. Imaged by AFM in aqueous solution and at room temperature, the isolated membrane samples appeared as patches of 4 nm height–the approximate thickness of a lipid bilayer–adsorbed a mica substrate (Fig 1). As reported previously [31], these membrane patches contained static microdomains with lateral dimensions in the range of 100–300 nm, protruding 1–2 nm from the patch surface, and with increased roughness compared to the surrounding membrane.

**Fig 2:**
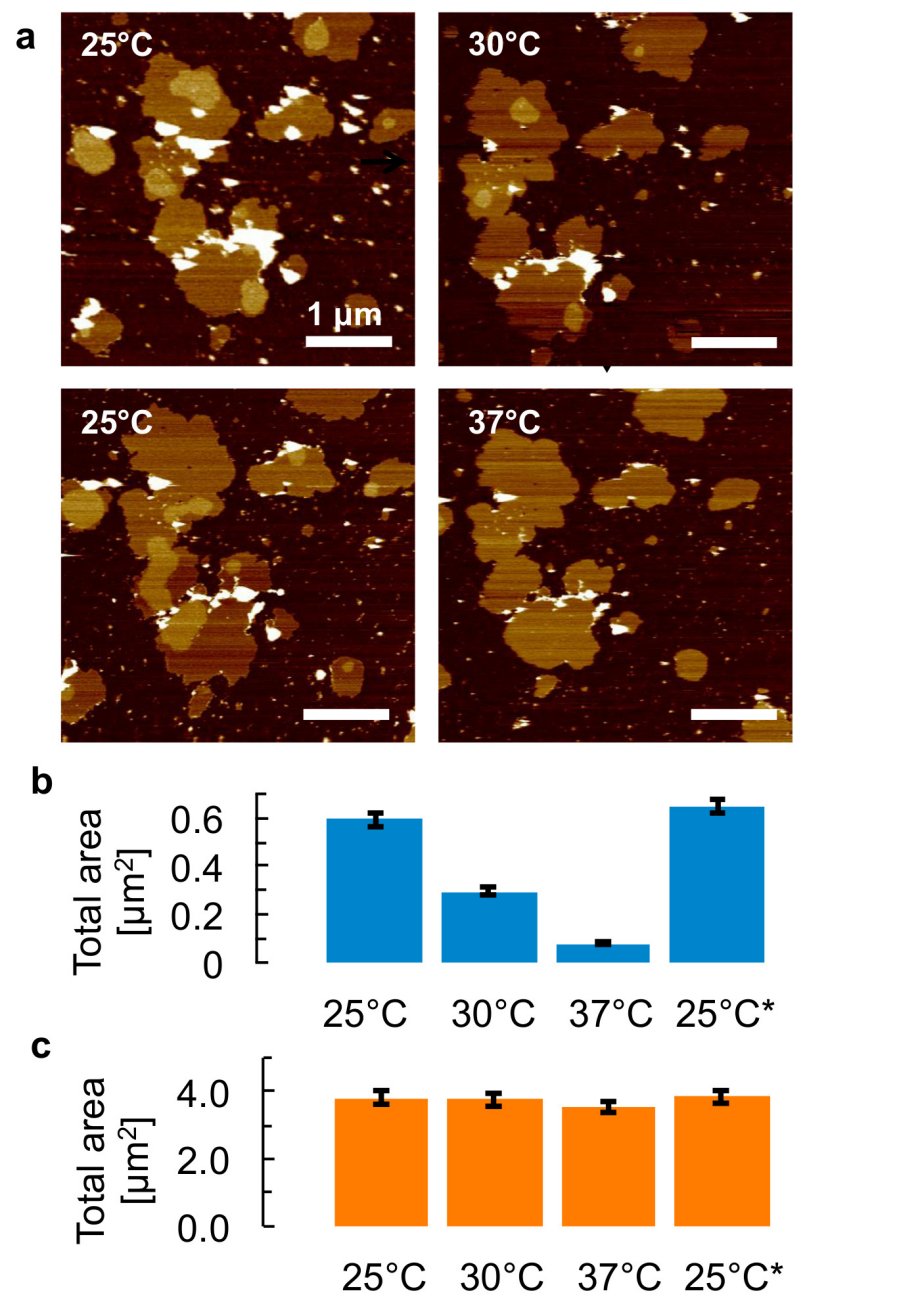
Reversible dissolution and formation of microdomains. **a**, AFM topography of isolated membrane samples in buffer solution, for a thermal cycle in which the temperature is first increased from 25°C to 30°C and then 37°C, followed by a decrease back to 25°C. Arrows indicate the sequence of the images. Vertical (color) scale for all AFM images: 9 nm, see also scale bar in Fig 1. **b**, Histogram of the surface area of the microdomains observed on the membrane patches in (a), demonstrating a shrinking of microdomains for higher temperatures and a full recovery at the end of the thermal cycle (25°C*). **c**, Histogram of the total surface area of all patches that are fully included within the frames displayed in (a), showing that the absolute changes in total patch area are small compared to the absolute changes in microdomain area over the thermal cycle. Error bars = 5% (see Methods).

Interestingly, the microdomains showed a marked temperature dependence, whereas the overall dimensions of the membrane patches appeared unchanged over the range of temperatures probed in this work. This is illustrated in Fig 2, which shows the same membrane patches imaged over a temperature cycle between 25°C and the physiological 37°C. In particular, when the temperature was increased to 30°C, the smaller microdomains (less than 100 nm in diameter) disappeared while the larger ones were reduced in size, and the total area of the microdomains in this frame reduced by more than a factor 2. On a further temperature rise to 37°C, microdomains could hardly be discerned any more. As demonstrated by subsequent cooling to 25°C, this process was reversible in the sense that the total microdomain area was identical at the beginning and the end of the 25°C—30°C—37°C—25°C cycle, though the sizes and positions of individual microdomains had changed. This indicates a dynamic nature of the individual microdomain constituents at higher temperatures. At 37°C, the remaining microdomains could also be observed to gradually change shape and position within the membrane patches, without changing in size (S2 Fig). Further, prolonged incubation at temperatures > 37°C did not affect the reformation of microdomains on cooling below physiological temperature (S3 Fig).

**Fig 3:**
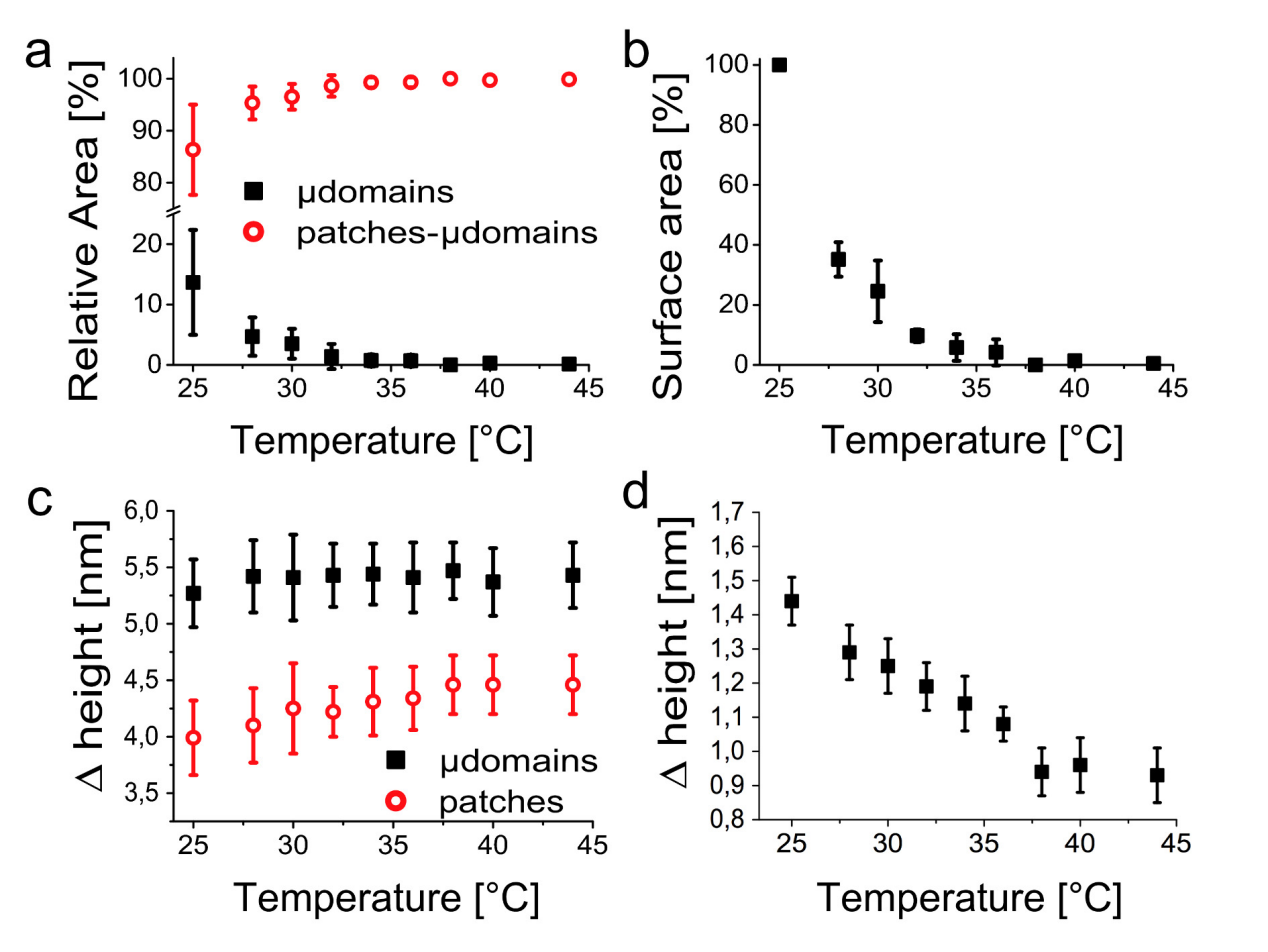
Temperature dependence of microdomain size and height. **a**, Plot of the relative areas of microdomains and membrane patches as a function of the temperature. **b**, Average of the surface area of individual microdomains as a function of temperature, normalized to the surface area of the respective microdomains at 25°C. **c**, Plot of the microdomain and membrane patch heights, both measured with respect to the mica, as a function of the temperature. **d**, Height of the microdomains measured with respect to the surrounding membrane patch surface, as a function of temperature. All data represent the mean ± SD obtained analyzing 16 different thermal cycles.

This behaviour was confirmed and further analysed in different experiments including in total 16 thermal cycles between a minimum of 23°C and a maximum of 46°C. Fig 3A reports the total relative areas on the membranes occupied by microdomains, as a function of temperature (100% = total area of all membrane patches observed in the AFM images). At 25°C, microdomains constituted about 15% of the total membrane area, a fraction that was rapidly reduced to a few % for temperatures between 32 and 36°C, and that was hardly distinguishable above 37°C. The same behaviour was observed using an alternative analysis, for which the total area of each microdomain at 25°C was defined as 100% and where the relative size of the microdomain was tracked as a function of temperature. Fig 3B shows the average relative areas for different microdomains in different experiments. We note that only in rare occasions, few and small microdomains persisted up to 44°C (see S4 Fig).

In protein-free lipid model membranes, liquid ordered (Lo) and liquid disordered (Ld) phases have been distinguished via the difference in their respective heights, which decreases on approaching a critical temperature from below, with the two phases becoming increasingly similar. Eventually, on raising the temperature, the observed domains disappear before the properties of the two phases become identical. Such effects can also be observed for the relative membrane heights in this work (Fig 3C). The microdomain height was constant at 5.4 ± 0.3 nm, as measured with respect to the mica substrate (where the errors here represent standard deviations). However, the height of the membrane patch (excluding the microdomains) increased from about 4.0 nm at 25°C to a constant 4.4 ± 0.3 nm above 37°C. Relative height measurements yielded more accurate values for the height difference between the microdomains and the surrounding membrane, decreasing from 1.4 ± 0.1 nm at 25°C to a constant 0.9 ± 0.1 nm above 37°C (Fig 3D). It should be emphasized that the height differences for *T* > 37°C represent only a tiny fraction of the membrane surface (~<1%, as explained above and visible in Fig 3A and 3B).

**Fig 4:**
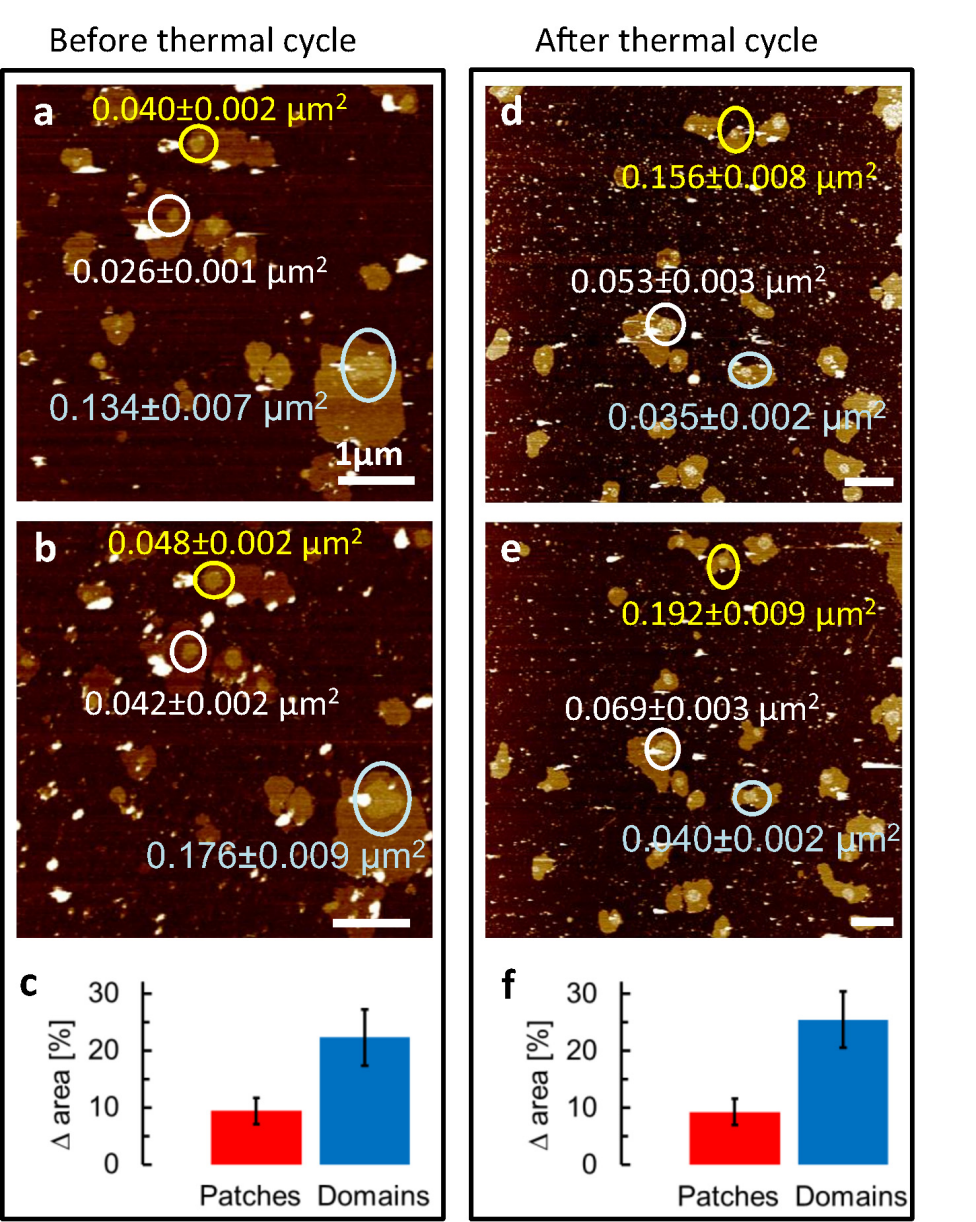
Immunolabelling of microdomains. AFM at room temperature to detect protein content in the microdomains, before (left) and after (right) a thermal cycle (25°C -37°C- 40°C- 25°C). **a**, Untreated membrane patches were incubated with anti flotillin-1 antibodies. **b**, After 60 min of incubation, the area of the microdomains protruding from the membrane patches had increased (see differently colored circles for individual examples). **c**, Histogram of the surface area increase for membrane patches and microdomains after 60 min antibody incubation. **d, e, f**, As (a, b, c), but for membrane patches that have undergone a thermal cycle prior to the immunolabeling. Student's t-test for the difference between relative increases of patch and microdomain areas: p<0.01 in both (c) and (f). Horizontal scale bar: 1 μm; vertical (color) scale: 9 nm in the four AFM images.

To verify for protein content in the observed microdomains, the purified and adsorbed membrane samples were imaged before and after 60min incubation with 50 μg/ml antibodies to target flotillin-1, a protein associated with lipid rafts [33], at room temperature. As observed previously [31], this immunolabeling lead to a relative increase of the microdomain areas that is significantly larger than that of the overall membrane patches (Fig 4A–4C). Importantly, this increase was the same irrespective of whether the immunolabeling was performed before or after a thermal cycle in which the temperature had been raised to 40°C (thermal cycle 25°C-37°C-40°C-25°C; Fig 4D–4F). This demonstrates that the protein content of the microdomains is recovered after dissolution and reformation of the microdomains, and thus confirms the overall reversibility of the temperature-induced changes in the microdomains.

## Discussion

There is increasing evidence that a wide range of membrane-associated processes are regulated by the formation of microdomains in the cell membrane [4], with molecular contents and structure that differ from the surrounding membrane. This AFM study demonstrates the presence of microdomains in detergent-resistant membranes that were isolated from human breast cancer cells [31], similar in size to those observed in isolated erythrocyte membranes at room temperature [32]. The microdomains protruded 1–2 nm from the surrounding membrane, suggesting a locally enhanced lipid order [9], and with larger roughness indicating significant protein content (Fig 1). The high spatial resolution of AFM enabled straightforward visualization of the microdomains in spite of their size being at or below the optical diffraction limit. Importantly, the AFM measurements allowed for an accurate determination of the microdomain area as a function of temperature, as well as of the height difference (at Ångström resolution) between the microdomains and the surrounding membrane, which can be considered as a label-free measure of lipid order [9, 34].

AFM can also be used to determine the phase behaviour of supported lipid membranes as a function of temperature [35]. Compared to free-standing membranes of equal composition, supported lipid membranes on mica (as studied in this work) have been reported to show more complicated phase behaviour due to decoupling between the upper and lower lipid leaflets of the bilayer [36], though other studies showed that gel transition temperatures for DMPC (24°C) were only shifted by +4°C due to the presence of the mica substrate [37, 38], whereas the 42°C transition temperature for DPPC (in a temperature range that may be more relevant to this work) agreed to within ±1°C for free-standing and mica-supported membranes [39].

The detergent-resistant membranes here were extracted using 1% Triton X-100, and subsequently separated by ultracentrifugation on 5%–30% sucrose density gradient, following the classical isolation methods for lipid rafts. It has been suggested that treatment of cells with Triton X-100 generates or promotes unphysiological clusters of raft lipids [40], though other, more recent data indicate that Triton X-100 does not induce domain formation, and only affects the domain size by coalescence of pre-existing domains [41]. As a consequence, the absolute domain sizes in our experiments may be overestimates compared to those in the cell membrane.

The abundance of detergent-resistant membranes has also been successfully correlated with phase separation in cell-membrane derived giant plasma membrane vesicles [42]. The detergent-resistant membranes may thus be interpreted as representing cholesterol-enriched and supposedly more ordered domains in cell membranes. The results presented here indicate that the detergent-resistant membrane fractions themselves can also show spatially segregated phases in the form of microdomains. A possible interpretation of our data is that the detergent-resistant membranes represent insoluble lipid domains in the cell membrane at the isolation temperature of 4°C, which on approaching physiological temperature loose much of their ordered nature.

In the detergent-resistant membranes from human breast cancer cells, the presence of microdomains was strongly temperature dependent (Fig 2), showing an order-of-magnitude drop in their total surface area between room temperature and the physiological 37°C, consistent with earlier observations on, e.g., giant plasma membrane vesicles [11, 12, 42]. On subsequent cooling to room temperature, the total microdomain area returned to its value before the temperature rise (i.e., is reversible). This indicates a dissolution of microdomain components in the surrounding membrane on raising the temperature, followed by (re)nucleation and growth on the subsequent temperature drop. Once dissolved, the microdomain constituents were freely moving within the membrane, as can be concluded from the observation that individual microdomains appeared with different sizes and shapes and at different locations before and after the thermal cycle.

On further quantification and averaging over different experiments, it could be shown that the microdomains were nearly fully dissolved at about 37°C (Fig 3A and 3B). This is a remarkable result, as it indicates that the difference between the microdomains and the surrounding membrane disappeared near the physiological temperature for these membranes, which implies that microdomains can be most easily formed and dissolved in the membranes, with possible implications for cell signalling, responding to intra- or extracellular triggers.

This behaviour is qualitatively similar to that observed for the phase transition between Lo and Ld phases in model membranes of ternary lipid mixtures including cholesterol [9]. This similarity was further verified by measuring the heights of and height difference between the microdomains and the surrounding membrane (Fig 3C). Since the Lo phase is a laterally condensed phase, it has a lower area per molecule and the lipid bilayer is higher (thicker). On increasing the temperature, one may expect a decrease in lipid order and compaction, leading to a decreasing height. In the isolated plasma membranes, however, the microdomain height did not show any significant temperature dependence. On the other hand, the surrounding membrane showed a gradual thickening with increasing temperature. This can be attributed to mixing of the microdomain components into the surrounding, presumably Ld membrane on increasing temperature.

Above ~37°C, a small fraction (~<1%) of the membranes remained organized in microdomains, but there was no further decrease in the height difference between the microdomains and the surrounding membrane. This suggests that these microdomains represent a chemically different subset with a higher transition temperature, or that a few microdomains have been trapped in non-equilibrium states, possibly because of interaction with the substrate and possibly indicating a high local protein content.

Immunolabelling with anti flotillin-1 (Fig 4) gave an indication of protein contents in the microdomains, and–because the association of flotillin-1 with lipid rafts [33]–suggested an identification of the microdomains as lipid rafts. As the labelling was equally successful before and after the thermal cycle, it follows that the protein also took part in the reformation of the microdomains on cooling below 37°C.

Taken together, these results indicate that detergent-resistant membranes from MDA-MB-231 human breast cancer cells undergo a reversible transition from a more to a less ordered state near physiological temperature, such that small changes in composition and intermolecular interactions may lead to large changes in membrane structure [34, 43]. This behaviour in the isolated, detergent-resistant membranes is in close agreement with liquid-disordered/liquid-ordered transitions that have been previously observed in lipid model membranes [9], but including the presence of a raft-associated protein. It is interesting to extrapolate this behaviour in the detergent-resistant membranes to the native cell membrane, with the caveat that such an extrapolation remains rather contentious: It would present a mechanism by which the plasma membrane may modulate its local morphology with corresponding impact on cell signalling and protein sorting processes.

## Supporting Information

S1 Fig Western blot analysis for flotillin-1. Western blot for the different fractions of the isolated membranes. Fraction 5 was used for the AFM analysis in this work, and–unlike the higher-density fractions (7–11, see also Material and Methods)–showed the presence of lipid-raft marker flotillin-1 (“Flot-1”) and of H. Ras, a small GTPase associated with microdomains. The membrane protein Clathrin was used as a negative marker. All fractions were separated by SDS-PAGE (10% polyacrylamide gel) and transferred onto a polyvinylidene difluoride (PVDF) membrane overnight then blocked in blocking buffer consisting of 5% (w/v) dried non-fat milk in Tris-buffered saline (T-TBS: 10mM Tris/HCl, pH 7.5, 150mM NaCl, 0.1% (v/v) Tween 20) at room temperature for 1h. The blots were treated with primary antibodies diluted 1:200 in blocking buffer at room temperature for 2h, washed with T-TBS and incubated with the proper secondary antibody in blocking buffer at room temperature for 1h. The protein bands were visualized using ECL reagents (PerkinElmer, USA).
(TIF)

S2 Fig Stability of microdomains at 37°C. AFM topography of isolated membrane samples in buffer solution, recorded over a 3hr time span at 37°C, showing roughly the same area with a microdomain that, while mobile, retains a constant surface area over time. Vertical (color) scale: 9 nm.
(TIF)

S3 Fig Prolonged incubation at higher temperatures. AFM topography of isolated membrane samples in buffer solution, in the same area, showing the effect of prolonged incubations at temperatures >37°C (thermal cycle 37°C—40°C -46°C -25°C). The microdomains reform at lower temperatures also after longer incubations at temperatures > 37°C. Vertical (color) scale: 9 nm.
(TIF)

S4 Fig Microdomain at 44°C. AFM topography of isolated membrane samples in buffer solution at 44°C, showing a rare case of a microdomain that persisted at temperatures >37°C. Vertical scale: 9 nm.
(TIF)
