# Supplementary figures and images for: Reversible Dissolution of Microdomains in Detergent-Resistant Membranes at Physiological Temperature
Source: PLoS One. 2015 Jul 6;10(7):e0132696. doi: 10.1371/journal.pone.0132696 (PMC4493071; doi:10.1371/journal.pone.0132696)

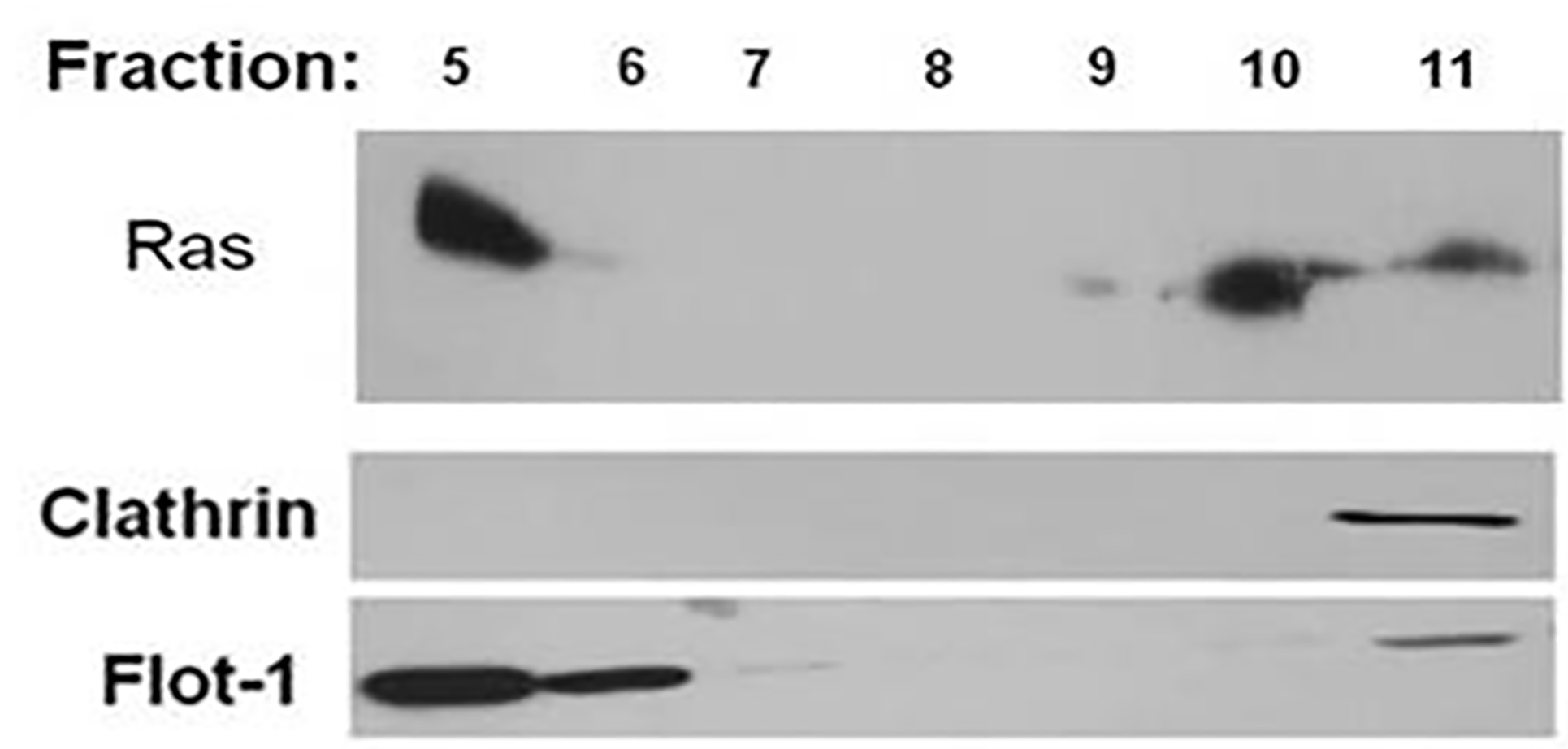

Supplement: S1 Fig — Western blot for the different fractions of the isolated membranes. Fraction 5 was used for the AFM analysis in this work, and–unlike the higher-density fractions (7–11, see also Material and Methods)–showed the presence of lipid-raft marker flotillin-1 (“Flot-1”) and of H. Ras, a small GTPase associated with microdomains. The membrane protein Clathrin was used as a negative marker. All fractions were separated by SDS-PAGE (10% polyacrylamide gel) and transferred onto a polyvinylidene difluoride (PVDF) membrane overnight then blocked in blocking buffer consisting of 5% (w/v) dried non-fat milk in Tris-buffered saline (T-TBS: 10mM Tris/HCl, pH 7.5, 150mM NaCl, 0.1% (v/v) Tween 20) at room temperature for 1h. The blots were treated with primary antibodies diluted 1:200 in blocking buffer at room temperature for 2h, washed with T-TBS and incubated with the proper secondary antibody in blocking buffer at room temperature for 1h. The protein bands were visualized using ECL reagents (PerkinElmer, USA). (TIF) [file pone.0132696.s001.tif]

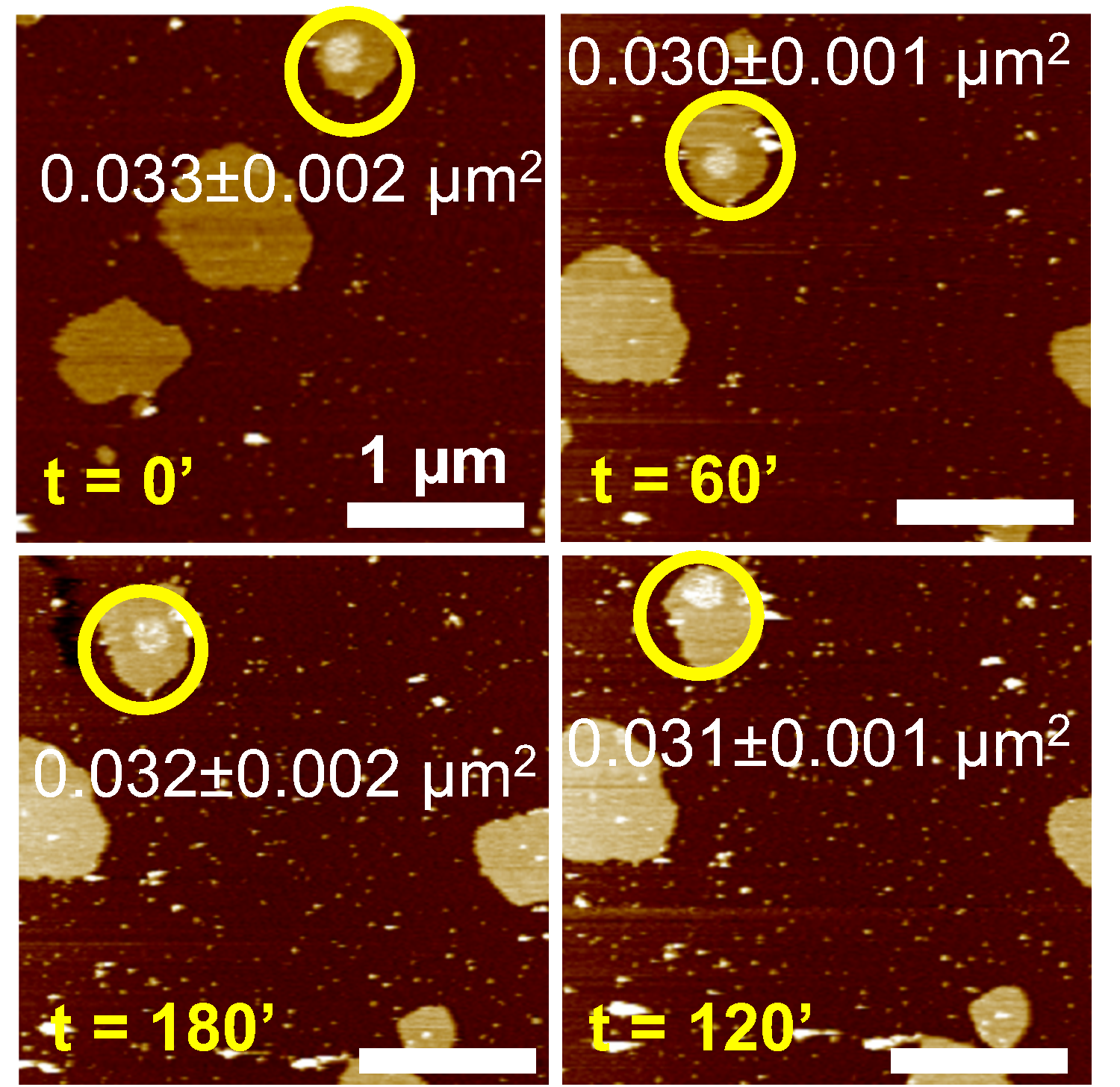

Supplement: S2 Fig — AFM topography of isolated membrane samples in buffer solution, recorded over a 3hr time span at 37°C, showing roughly the same area with a microdomain that, while mobile, retains a constant surface area over time. Vertical (color) scale: 9 nm. (TIF) [file pone.0132696.s002.tif]

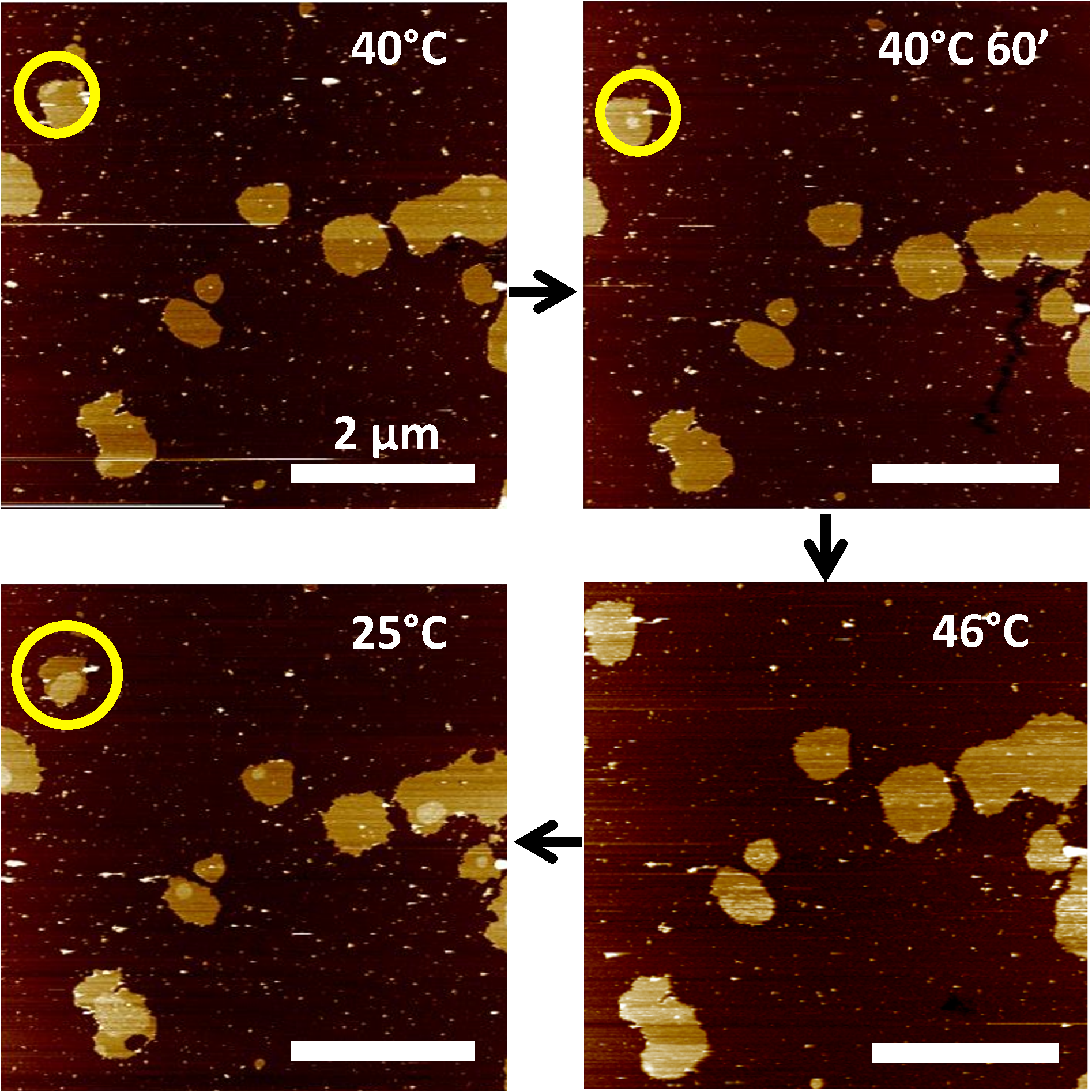

Supplement: S3 Fig — AFM topography of isolated membrane samples in buffer solution, in the same area, showing the effect of prolonged incubations at temperatures >37°C (thermal cycle 37°C—40°C -46°C -25°C). The microdomains reform at lower temperatures also after longer incubations at temperatures > 37°C. Vertical (color) scale: 9 nm. (TIF) [file pone.0132696.s003.tif]

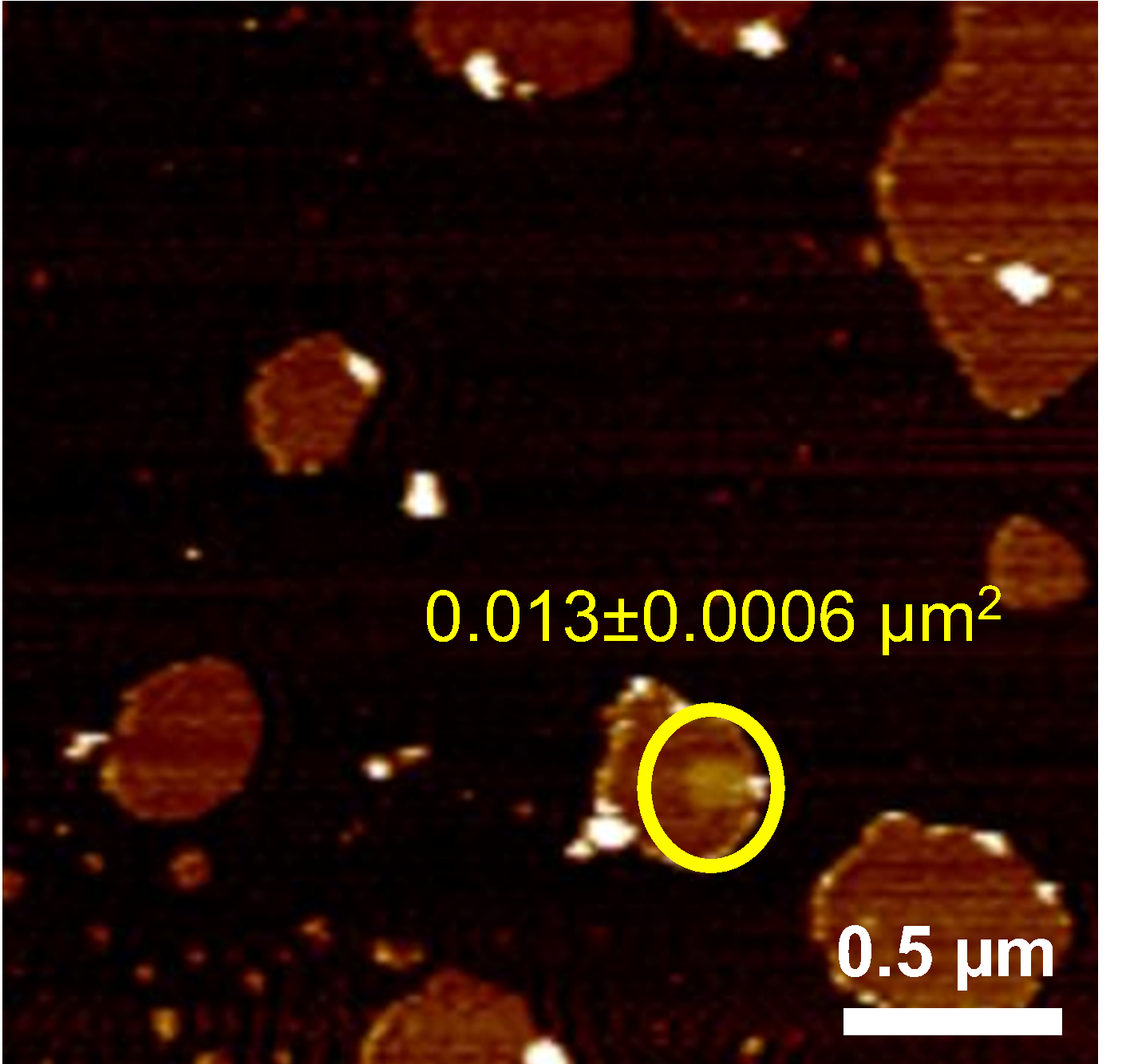

Supplement: S4 Fig — AFM topography of isolated membrane samples in buffer solution at 44°C, showing a rare case of a microdomain that persisted at temperatures >37°C. Vertical scale: 9 nm. (TIF) [file pone.0132696.s004.tif]
